# Extending Mathematical Frameworks to Investigate Neuronal Dynamics in the Presence of Microglial Ensheathment

**DOI:** 10.1007/s11538-025-01438-w

**Published:** 2025-04-04

**Authors:** Nellie Garcia, Silvie Reitz, Gregory Handy

**Affiliations:** School of Mathematics, University of Minnesota, 127 Vincent Hall 206 Church St. SE, Minneapolis, MN 55455 USA

**Keywords:** Particle diffusion, Exponential integrate-and-fire neurons, Linear response theory, Gamma oscillations

## Abstract

Recent experimental evidence has shown that glial cells, including microglia and astrocytes, can ensheathe specific synapses, positioning them to disrupt neurotransmitter flow between pre- and post-synaptic terminals. This study, as part of the special issue “Problems, Progress and Perspectives in Mathematical and Computational Biology,” expands micro- and network-scale theoretical frameworks to incorporate these new experimental observations that introduce substantial heterogeneities into the system. Specifically, we aim to explore how varying degrees of synaptic ensheathment affect synaptic communication and network dynamics. Consistent with previous studies, our microscale model shows that ensheathment accelerates synaptic transmission while reducing its strength and reliability, with the potential to effectively switch off synaptic connections. Building on these findings, we integrate an “effective” glial cell model into a large-scale neuronal network. Specifically, we analyze a network with highly heterogeneous synaptic strengths and time constants, where glial proximity parametrizes synaptic properties. This parametrization results in a multimodal distribution of synaptic parameters across the network, introducing significantly greater variability compared to previous modeling efforts that assumed a normal distribution. This framework is applied to large networks of exponential integrate-and-fire neurons, extending linear response theory to analyze not only firing rate distributions but also noise correlations across the network. Despite the significant heterogeneity in the system, a mean-field approximation accurately captures network statistics. We demonstrate the utility of our model by reproducing experimental findings, showing that microglial ensheathment leads to post-anesthesia hyperactivity in excitatory neurons of mice. Furthermore, we explore how glial ensheathment may be used in the visual cortex to target specific neuronal subclasses, tuning higher-order network statistics.

## Introduction

Recent advancements in experimental techniques and approaches in neuroscience have expanded the field beyond the traditional excitatory-inhibitory framework, revealing that various neuronal subtypes and glial cells significantly influence cortical dynamics. Instead of being a homogeneous class, 80% of interneurons are divided into three subtypes: parvalbumin (PV)-, somatostatin (SOM)-, and vasointestinal peptide (VIP)-expressing neurons, each with distinct properties (Pfeffer et al. 2013; Jiang et al. 2015; Karnani et al. 2016; Tremblay et al. 2016). Additionally, astrocytes and microglia, types of glial cells, have been found to closely ensheathe synapses, positioning them to fine-tune synaptic properties (Ventura and Harris 1999; Chever et al. 2016; Haruwaka et al. 2024). These findings highlight the need for theoretical frameworks that incorporate cellular heterogeneity and offer deeper insights into how the brain utilizes this diversity in cortical computations.

Glial cells are thought to engage in bidirectional communication with neurons through several pathways. Experimental evidence supports their involvement in neurotransmitter clearance from the synaptic cleft, alterations of extracellular ion concentrations, the release of neuroactive substances via gliotransmission, and synaptic pruning (Tzingounis and Wadiche 2007; Wake et al. 2009; Paolicelli et al. 2011; Covelo and Araque 2018). Existing computational models have explored some of these functions, focusing on the concept of the “tripartite synapse,” which includes the pre- and post-synaptic terminals, as well as a neighboring astrocyte (Araque et al. 1999). These models, which primarily depend on gliotransmission, have demonstrated how astrocytes modulate network behavior, including influencing long-term potentiation/depression and modulating thresholds for focal seizure generation (Reato et al. 2012; Amiri et al. 2012; Pittà and Brunel 2016).

However, these models assume that the strength of astrocytic influence does not vary from synapse to synapse, despite evidence suggesting that astrocyte proximity to synapses, referred to here as ensheathment strength, is heterogeneous, varying across brain regions and disease states such as epilepsy and Alzheimer’s disease (Lippman et al. 2008; Coulter and Steinhauser 2015; Matias et al. 2019; Price et al. 2021). Furthermore, recent experimental work by Haruwaka et al. (2024) demonstrated that changes in microglial ensheathment alone were sufficient to induce changes in network firing rates by shielding inhibitory synapses and disrupting neurotransmitter flow. This highlights a more subtle effect that both astrocytes and microglia can have on a network-an effect that does not rely on the sometimes controversial mechanism of gliotransmission (Nedergaard and Verkhratsky 2012; Fujita et al. 2014; Haydon and Nedergaard 2015). A recent model by Handy and Borisyuk (2023) investigated this effect with a detailed microscale model of synapses and derived an “effective” glial ensheathment framework that could be incorporated into large-scale neural networks. This computational study suggested that changes in synaptic strength and time course brought on by glial ensheathment could induce shifts in neural network synchrony. However, this work has a few shortcomings. First, the level of heterogeneity remained constrained, as the authors considered a binary level of ensheathment (each synapse was either ensheathed or not), while experiments show a distribution of ensheathment levels (Haruwaka et al. 2024). Second, their network model consisted of only two populations-excitatory and inhibitory neurons-and they primarily focused on the ensheathment of outgoing excitatory connections. Finally, it was left as an open question whether this heterogeneity could be accurately accounted for in a simplified mean-field model.

In this work, we aim to overcome these shortcomings by improving upon the “effective” glial model and incorporating it into a model of mouse primary visual cortex (V1), which accounts for the additional interneuron subclasses of PV and SST. Recent experimental and modeling work has suggested that recurrent neuronal networks utilize these different inhibitory neurons to robustly perform complex cortical computations, such as tuning local and global oscillatory dynamics, recovering neuronal gain after injury, and shaping responses to visual stimuli during locomotion (Veit et al. 2023; Kumar et al. 2023; Dipoppa et al. 2018). This has led to the conjecture that there is a strong division of labor between interneurons, with different interneuron subtypes serving distinct roles in network activity and computation (Bos et al. 2020). With this hypothesis in mind, it remains an open question whether glial ensheathment can modulate network dynamics in a similar fashion by targeting specific interneuron subtypes, motivating our choice of network configuration. Specifically, we will examine whether glial ensheathment can modulate the strength and synchrony of visually induced gamma oscillations, which are rhythms thought to promote the contextual synthesis of visual percepts (Fries 2009).

In addition to constructing a spiking neuronal network that accounts for the heterogeneity induced by glial cells ensheathing specific synapses, we seek to extend linear response theory and develop a mean-field approximation that captures these results. Linear response theory describes how a cell’s stationary firing rate responds to weak perturbations (Risken 1996; Lindner and Schimansky-Geier 2001; Brunel et al. 2001). It relies on the cell’s susceptibility function, a linear approximation of the neuron’s response to an input that implicitly depends on model parameters. Previous studies (Lindner et al. 2005; Trousdale et al. 2012) have extended this framework to networks by treating the synaptic inputs a cell receives as weak perturbations. This theory has been leveraged to investigate how neuronal firing rates and correlations respond to stochastic fluctuations (e.g., noisy inputs and synaptic connections) in their environment (Doiron et al. 2004; Lindner et al. 2005; Trousdale et al. 2012; Ocker et al. 2015). Recently, Veit et al. (2023) applied such theory to a network to investigate how these interneurons sub tune the strength of gamma rhythms. After extending this theory to include glial ensheathment, we will use it to perform an expansive parameter sweep, exploring the range of effects that glial ensheathment can have on network dynamics.

The paper proceeds with the following structure. First, we detail the modeling frameworks (Sect. 2), including the microscale model of glial ensheathment and the network model. In Sect. 2.1, we refine the “effective” glial ensheathment model presented in Handy and Borisyuk (2023) to fit a more realistic synaptic kernel and achieve greater accuracy across a range of ensheathment strengths. Section 2.2 then details the network model, extending previous works by incorporating multiple neuronal subtypes and varying levels of glial ensheathment in a spiking network and its corresponding mean-field approximation. We then present key results in Sect. 3, where we replicate the experimental findings of Haruwaka et al. (2024), demonstrating that ensheathment of inhibitory synapses leads to hyperexcitability. We go beyond those results to explore how glial ensheathment affects correlations, particularly in modulating the strength and synchrony of gamma rhythms. After validating the match between spiking simulations and the mean-field approximation, we use our theory to conduct a large parameter sweep, comparing the effects of ensheathing PV neurons with those of SST neurons. Finally, in Sect. 4, we discuss these key results, model limitations, and future directions.

## Models and Linear Response Theory

### Microscale Model of Glial Ensheathment

Haruwaka et al. (2024) observed that microglia ensheathed individual inhibitory synapses to varying degrees, including no contact, low contact, enwrapment, and complete engulfment, and that the overall level of ensheathment changed before and after anesthesia induced by the administration of isoflurane. Imaging data suggested that this ensheathment “shielded” pre-synaptic terminals, preventing neurotransmitters from diffusing across the synaptic cleft to the adjacent post-synaptic terminal.

However, due to the small scale of the synaptic cleft, how various levels of glial ensheathment tune synaptic interactions between pre- and post-synaptic partners remains an open question. While our primary goal in this paper is to observe how these effects shape network dynamics, we must first understand this microscale effect to develop a clear approach for incorporating glial ensheathment into such a network model. To address this problem, we utilize the diffusion with recharging traps (DiRT) process (Handy et al. 2018, 2019; Handy and Lawley 2021) to model this shielding process at varying levels of glial cell ensheathment. This modeling framework has previously been used to develop an “effective" glial model that shaped synaptic interactions governed by a decaying exponential (Handy and Borisyuk 2023). We aim to refine this result by fitting a more appropriate synaptic interaction kernel to the stochastic simulations and generalizing the results to account for additional geometries of glial ensheathment.

#### Diffusion with Recharging Traps in an Idealized Synaptic Cleft

We start by considering $N_\text {NT}$ neurotransmitters diffusing within an idealized synapse, governed by the equation

1 $$\begin{aligned} dX_k(t) = \sqrt{2D}\cdot dW_k(t), k = 1,...,N_\text {NT} \text { for } X_k(t) \in \left( \Omega ^\text {cleft} \cup \Omega ^\text {extra}\right) , \end{aligned}$$

where $X_k(t)$ denotes the location of the neurotransmitter, $W_k(t)$ represents independent Wiener processes, and *D* is the diffusion coefficient. The synaptic cleft is modeled as a two-dimensional domain,

$$\begin{aligned} \Omega ^\text {cleft} = [0,c_w]\times [0,c_h], \end{aligned}$$

where $c_w$ is the width and $c_h$ is the height of the cleft. At $t = 0$, all $N_\text {NT}$ neurotransmitters are released simultaneously at ($c_w/2,c_h$).

The boundary of the ensheathing glial cell is considered to be perfectly absorbing and can protrude into the cleft in either a symmetric or non-symmetric fashion. For the symmetric case, the boundary is defined to be

$$\begin{aligned} \partial \Omega _\text {glial}^\text {sym} = \left\{ (x,y)|x=c_w\cdot (\phi /2) \text { and } x = c_w \cdot (1-\phi /2)\right\} , \end{aligned}$$

where $\phi \in [-1, 1]$ denotes the fraction of the synaptic cleft obstructed by glial protrusion. For the non-symmetric case, we allow the glial cell to only protrude from the right-hand side of the domain, with the left-hand side fixed sufficiently far away as to not significantly impact neurotransmitter trajectories within the cleft, and so

$$\begin{aligned} \partial \Omega _\text {glial}^\text {ns} = \left\{ (x,y)|x=-1 \text { and } x = c_w \cdot (1-\phi /2)\right\} . \end{aligned}$$

**Fig. 1:**
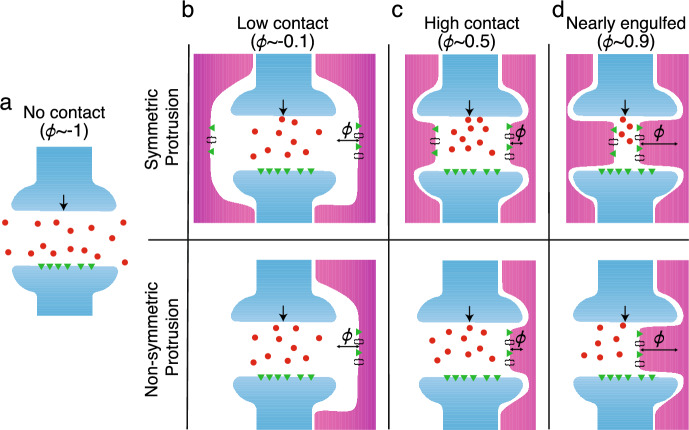
Schematics of the synaptic cleft, consisting of the pre- and post-synaptic terminals (blue) with different levels ($\phi$) of glia (magenta) ensheathment with either symmetric (top row) or non-symmetric protrusion (bottom row). Neurotransmitters (red circles) are released from the pre-synaptic terminal (black arrow) and diffuse freely within the domain. They are removed from the domain by interacting with neurotransmitter receptors and transporters (e.g., N-methyl D-aspartate receptor, excitatory amino acid transporter), which are denoted as green triangles and black brackets, respectively. **a** no contact ($\phi \sim -1$), **b** low contact ($\phi \sim -0.1$), **c** high contact/enwrapped ($\phi \sim 0.5$), and **d** nearly engulfed ($\phi \sim 0.9$) (Color figure online)

Importantly, in both cases, $\phi = 1$ denotes the complete ensheathment of the synapse, since the release site of neurotransmitters is entirely blocked by the protruding glial cell, preventing any neurotransmitters from entering the cleft. Further, negative values of $\phi$ indicate that there exists space between the glial cell and the synaptic cleft, in which case the domain outside of the cleft is referred to as the extracellular space ($\Omega ^\text {extra}$). Figure 1a illustrates a schematic of this domain when there is no glial ensheathment, while Fig. 1b–d shows it for various levels of glial ensheathment for both symmetric (top) and non-symmetric (bottom) protrusion.

Along the bottom boundary of $\Omega ^\text {cleft}$ lies the postsynaptic density, where $N_\text {rec}$ partially absorbing postsynaptic receptors of equal size are placed according to

$$\begin{aligned} \partial \Omega _\text {rec}^\text {cleft} = \left\{ (x, y) \mid y = 0, x \in [\text {psd}_l, \text {psd}_r]\right\} , \end{aligned}$$

where $\text {psd}_l$ and $\text {psd}_r$ denote the left and right endpoints of the postsynaptic density. The probability of a particle being absorbed upon making contact is given by

2 $$\begin{aligned} \text {Probability of absorption} = \frac{K\sqrt{\pi }}{\sqrt{D}}\sqrt{\Delta t}, \end{aligned}$$

where $\Delta t$ is the time step of the diffusion model and *K* is the absorption rate. If a molecule hits an available receptor, it binds to the receptor with the probability given by Eq. 2. After a successful absorption, the receptor becomes activated and switches to a transitory refractory state, during which it is unable to bind additional molecules. If a receptor is in the refractory state, any neurotransmitter that hits it is reflected back into the domain. The time spent in this refractory state is modeled as exponentially distributed, with a mean $\tau _r > 0$. Once the receptor exits the refractory state, the bound neurotransmitter is removed from the system and the receptor returns to being partially absorbing. The remaining boundaries (i.e., the remaining portions of the pre- and post-synaptic terminals) are considered reflecting.

Compared to other frameworks that consider diffusing particles searching for a target (Doering 2000; Lawley et al. 2015; Bressloff and Lawley 2015; Bressloff and Kim 2019; Bressloff 2020a, b; Gomez and Lawley 2024), the boundary conditions here depend on the paths of individual particles, significantly complicating the mathematical analysis of this problem. As a result, extending relevant asymptotic formulas, such as those describing the distribution of particle arrival times at receptors, derived under the assumption of particle independence to account for these statistical correlations is not straightforward and is left for future work. Instead, we rely on numerical simulations to gain insight into the time course of the number of activated receptors as a function of time.

#### Numerical Details for the DiRT Simulations

We use the Euler–Maruyama method (Kloeden and Platen 1992) for simulating Eq. 1, implemented in a combination of C and MATLAB (2023). All parameters corresponding to this ensheathment simulation can be found in Table 1. While the units used in this idealized synapse are arbitrary, they were previously parameterized in Handy et al. (2018, 2019) and Handy and Borisyuk (2023) to reflect relative dimensions of synaptic clefts (i.e., it is wider than its height), the rates of the receptor kinetics (i.e., the diffusion coefficient of neurotransmitters is fast relative to the recharge rate of the receptor), as well as the ratio of neurotransmitters to receptors.

**Table 1:** Default parameter value for the DiRT model (arbitrary units)

Parameter	Default value	Description
$N_\text {NT}$	1000	Number of neurotransmitters released
$N_\text {rec}$	50	Number of postsynaptic receptors
$\tau _r$	0.1	Mean receptor recharge time
*K*	1	Absorption rate
*D*	1	Diffusion coefficient
$c_w$	1	Cleft width
$c_h$	0.1	Cleft height
$[{\text {psd}_{l}}, {\text {psd}_{r}}]$	[0.25 0.75]	Postsynaptic density location
$\phi$	$-1$ to 1	Fraction of the cleft blocked by protruding glial cell
$\Delta t$	$1\times 10^{-5}$	Diffusion time step

All parameter values are the same as Handy and Borisyuk (2023)

#### DiRT Simulations and “Effective” Glial Ensheathment Model

**Fig. 2:**
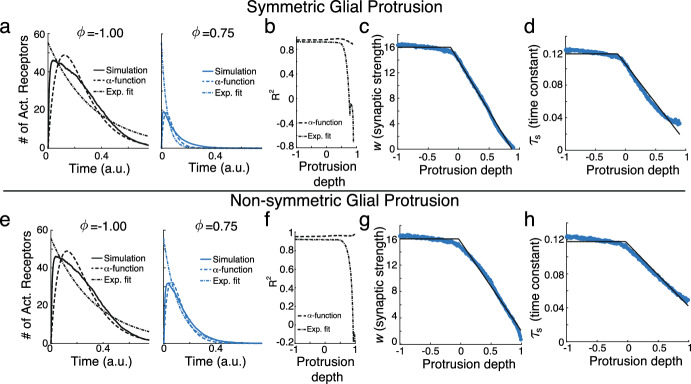
**a** DiRT simulation (solid line) of neurotransmitters diffusing in the synaptic cleft with symmetric glial protrusion for $\phi = -1$ (black) and $\phi = 0.75$ (blue) and corresponding fitting results for the $\alpha$-function (dashed) and exponential function (dot-dashed). **b** $R^2$ values for the function fit as a function of $\phi$ for the $\alpha$-function (dashed) and exponential fit (dot-dashed). **c** Fitted synaptic strengths, $w(\phi )$ for different protrusion amounts (blue dots) and the fitted piecewise function from Eq. 3 (black). **d** Same as **c**, except for the synaptic time constants $\tau _s(\phi )$. **e**–**h** Same as a-d but for neurotransmitters diffusing in the synaptic cleft with non-symmetric glial protrusion (Color figure online)

Simulations of this stochastic process demonstrate how the time course of the number of active receptors is influenced by symmetric glial ensheathment via changes in the protrusion parameter $\phi$ (Fig. 2a). We find that as $\phi \rightarrow 1$, both the maximum number of activated receptors and the time course of this activation significantly decrease. To further quantify this change, we fit the curves to $\alpha$-functions, a standard model of synaptic interactions (Trousdale et al. 2012),

$$\begin{aligned} \alpha (t,\phi )&= w(\phi )\cdot J(t,\phi ) \\&= w(\phi )\cdot \frac{t}{\tau _s(\phi )^2}\exp \left[ -\frac{t}{\tau _s(\phi )}\right] {\mathcal {H}}(t), \end{aligned}$$

where we have normalized the $\alpha$-function so that

$$\begin{aligned} \int ^\infty _{-\infty } J(t, \phi ) dt =1. \end{aligned}$$

The parameters for this non-linear function were fit by feeding in the results of the DiRT simulations, saved at every $10^{-4}$ time step, into MATLAB’s fitnlm function (MATLAB 2023).

In this formulation, $w(\phi )$ and $\tau _s(\phi )$ correspond to the synaptic strength and synaptic time constant, respectively, for different levels of $\phi$. Figure 2a illustrates that this function provides a reasonable fit (dashed line) for $\phi = -1$ and $\phi = 0.75$ when compared to the DiRT simulations (solid line) ($R^2$ = 0.951 and 0.929 for these two values of $\phi$, respectively).

Conducting simulations of this stochastic process for values of $\phi \in [-1, 1]$, discretizing the interval with a step size of 0.01 and only keeping simulation results where at least one neurotransmitter successfully finds a receptor, we observe that this fit consistently performs well, with an average $R^2$ values of 0.95 (Fig. 2b, dashed). Further, we find that $w(\phi )$ and $\tau _s(\phi )$ follow a piecewise linear pattern (Fig. 2c, d, blue circles). Specifically, for negative $\phi$, $w(\phi )$ and $\tau _s(\phi )$ remain constant, indicating that the neighboring glial cell has no effect on synaptic transmission. Both values then decrease linearly as $\phi$ increases towards 1. Assuming this general form, we estimate the transition point by fitting a piecewise linear function:

3 $$\begin{aligned} w(\phi ) = w^\text {max} + (w_s \cdot (\phi - \phi _w)) \cdot {\mathcal {H}}(\phi - \phi _w), \end{aligned}$$

to the simulated data points (Fig. 2c, d, black line). To perform this fit and estimate the values of $w^\text {max}$, $w_s$, and $\phi _w$, we fed in all of the fitted values of *w*, estimated at every 0.01 intervals of $\phi$, into MATLAB’s built-in fitnlm function (MATLAB 2023). We find $w^\text {max} = 16.024$, $w_s = -15.917$, and $\phi _w = -0.134$. Interestingly, this suggests that glial cells can influence synaptic transmission by simply being in the *proximity* of a synapse (i.e., direct contact is not necessary). Furthermore, $w(\phi ) \rightarrow 0$ as $\phi \rightarrow 1$. The fit for $\tau _s(\phi )$ is similar, except that $\tau _s(\phi ) \rightarrow \tau _s^\text {min} > 0$ as $\phi \rightarrow 1$. Interestingly, these qualitative results remain true for the case of non-symmetric glial protrusion (Fig. 2e–h), and, as the theoretical results from previous work investigating the DiRT model (Handy et al. 2018, 2019) suggest, are rather robust to changes in the underlying parameters.

Performing such detailed diffusion simulations of neurotransmitters is infeasible in the context of a large-scale spiking neuronal network, but Eq. 3 suggests an effective glial ensheathment model that can be readily implemented to account for changes in synaptic strength and time constant. Specifically, let $w_{ab}$ be the default (maximal) synaptic strength from neuron population *b* to *a*, $s_{en} \in [0,1]$ denote the degree of ensheathment (i.e., $s=0$ indicates no ensheathment, and $s=1$ indicates full engulfment), and $\textbf{1}_{ij}$ be an indicator function that equals 1 if the *ij* synapses is ensheathed. Approximating $w_s\approx - w_{ab}$ and substituting $s_{en} = \phi - \phi _w$ into Eq. 3 gives:

4 $$\begin{aligned} w_{ij} = w_{ab} \cdot \left( 1 - s_{en}\cdot \textbf{1}_{ij}\right) . \end{aligned}$$

The equation for $\tau _s^{ij}$ is similar, except we account for its minimal value, $\tau _s^{\min } = (1-\beta )\tau _s$ for $\beta \in (0,1)$, as $s_{en} \rightarrow 1$:

5 $$\begin{aligned} \tau _s^{ij}&= \tau _s^{\min } + \left( \tau _s^{ab} - \tau _s^{\min } \right) \cdot \left( 1 - s_{en}\cdot \textbf{1}_{ij}\right) \nonumber \\&= \tau _s \left( 1-\beta s_{en}\cdot \textbf{1}_{ij}\right) , \end{aligned}$$

Based off of our simulations, we found the ratio of $\tau _s^\text {min}$ to $\tau _s$ to be 0.4.

This model of an “effective” glial cell can be readily implemented into a large-scale network, as discussed below. While it appears to be similar to the results found in Handy and Borisyuk (2023), this model differs in two ways. First, we consider $\alpha$-functions for the synaptic interactions and explicitly fit the synaptic strength and time constants to the simulation results. Second, by accounting for $\tau _s^{\min }$, our model allows the maximal height of the synaptic interactions to vary as a function of protrusion. In the previous work, they considered exponential synapses and made a phenomenological argument that the height of this exponential stayed fixed as a function of protrusion. Figure 2a, e (dot-dashed) show the best-fit lines using this previous framework, which significantly under performs our updated model in terms of $R^2$ (Fig. 2b, f, dot-dashed), especially as $\phi \rightarrow 1$ (note: a negative $R^2$ value indicates that the model performs worse than a constant). Overall, the investigation performed here results in a model that more accurately captures how glial ensheathment makes synapses faster and weaker, while also demonstrating that it is robust to the geometry of glial protrusion.

### Mathematical Model of Spiking Network with Glial Ensheathment and Corresponding Linear Response Theory

In this work, we are interested in exploring how glial ensheathment can modulate average network statistics such as firing rates and correlations. To that end, in this section we develop a mean-field linear response theory that can be used for this purpose. After outlining details of the exponential integrate-and-fire network with glial ensheathment (Sect. 2.2.1), we derive a self-consistency relationship for the the average population steady-state firing rates and effective mean inputs (Sect. 2.2.2). Using this relationship, we then derive a formulas for the average power- and cross-spectrums (Sect. 2.2.3). We then note how these careful derivations differ from a more naïve approach (Sect. 2.2.4).

#### Exponential Integrate-and-Fire Network with Glial Ensheathment

We start by considering a general network architecture that our theory can be applied to, before imposing a more specific network structure governed by the underlying biology (see Sect. 2.3). Namely, we consider a network of *N* recurrently connected exponential integrate-and-fire (EIF) neurons with membrane potentials of the form

6 $$\begin{aligned} \tau _m \frac{dV_i}{dt} = -(V_i-E_L) + \psi (V_i) + I_i(t) + I_i^{ext}(t). \end{aligned}$$

Each neuron $i=1,...,N$ belongs to a population $a = 1,..., P$, where neurons within the same population share the same intrinsic spiking properties and connectivity rules. A neuron spikes when $V_i(t) \ge V_{th}$, after which its value is reset to $V_{re}$. It then undergoes a refractory period of length $\tau _\text {ref}$ in which $V_i(t)$ is held at $V_{re}$ throughout. Here, $E_L$ denotes the leak reversal potential and $\psi (V)$ represents the spike-generating current, which takes the form

$$\begin{aligned} \psi (V) = \Delta _T \exp \left[ \frac{V-V_T}{\Delta _T}\right] . \end{aligned}$$

Synaptic interactions are modeled as

$$\begin{aligned} I_i(t) = \sum _i W_{ij}\cdot (J_{ij}*y_j)(t), \end{aligned}$$

where the spike train from neuron *j* is the point process $y_j(t) = \sum _k \delta (t-t_{j,k})$ and $*$ denotes convolution. Following the work from our microscale model in Sect. 2.1.3, synaptic interactions are modeled by delayed $\alpha$-functions of the form

$$\begin{aligned} J_{ij}(t) = \frac{t-\tau _{\text {delay},j}}{(\tau _s^{ij})^2}\exp \left[ -\frac{t-\tau _{\text {delay},j}}{\tau _s^{ij}}\right] {\mathcal {H}}(t-\tau _{\text {delay},j}). \end{aligned}$$

The synaptic weights are given by

$$\begin{aligned} W_{ij} = {\left\{ \begin{array}{ll} w_{ij} & \text {if { j} is connected to { i}} \\ 0 & \text {otherwise} \end{array}\right. }. \end{aligned}$$

Based on the results of the microscale ensheathment model (see Eqs. 4 and 5), the weights $w_{ij}$ and time constants $\tau ^{ij}_s$ are adjusted according to the ensheathment strength. To capture variability across individual synapses, we allow the ensheathment strength to vary over *m* discrete levels. Specifically, let $s_{en}^k\in [0,1]$ denote the $k = 1, 2,..., m$ possible levels of ensheathment strengthens. Then, for a synaptic connection from neuron *j* to neuron *i*, let $\textbf{1}_{ij}^k$ be an indicator function that equals 1 if the *ij* synapse is ensheathed at level *k* and zero otherwise. We can then write the synaptic strength and time constant for synapse *ij* as

$$\begin{aligned} w_{ij}&= w_{ab}\cdot \left( 1 - \sum _k s_{en}^k \cdot \textbf{1}_{ij}^k\right) , \text { and} \\ \tau ^{ij}_s&= \tau _s\left( 1 - \sum _k \beta s_{en}^k \cdot \textbf{1}_{ij}^k\right) . \end{aligned}$$

Finally, the last term of Eq. 6, $I_i^{ext}(t)$, represents the external drive to the network, which we decompose into independent and shared components,

$$\begin{aligned} I_i^{ext}(t) = I_i^{ind}(t) + I_{shared}(t). \end{aligned}$$

The independent component represents synaptic connections originating from outside the circuit we are considering. These connections can be either a fixed background input or variable feedforward inputs from outside the network (e.g., those relating to locomotion or a visual stimulus). Assuming all of these inputs arrive as Poisson spike trains, we can capture their impact on the membrane potential via a diffusion approximation and Campbell’s theorem (Kingman 1993), resulting in

$$\begin{aligned} I_i^{ind}(t) = \mu _{i,bg} + \mu _{i,\text {ffwd}} + \sqrt{(\sigma _{i,bg}^2 + \sigma _{i,\text {ffwd}}^2)2\tau _m}\cdot \xi _i(t), \end{aligned}$$

where $\mu _{i,bg}$ and $\mu _{i,\text {ffwd}}$ are the average mean inputs (i.e., drift) of these inputs, $\sigma _{i,bg}$ and $\sigma _{i,\text {ffwd}}$ capture the variability (i.e., diffusion coefficient) of these inputs, and $\xi _i(t)$ is a zero mean, delta-correlated, $\langle \xi _i(t),\xi _i(t')\rangle = \delta (t-t')$, Gaussian white noise term. The shared input captures additional shared variability felt across the circuit, such as the concentration of diffusive neuromodulators in the extracellular space, and is taken to be

$$\begin{aligned} I_{shared}(t) = \sigma _{gl} \sqrt{2\tau _m}\cdot \eta _{gl}(t), \end{aligned}$$

where the global noise process is $\eta _{gl}(t)$ is primarily considered to be a zero mean, delta-correlated Gaussian white noise (Veit et al. 2023). However, we note that our corresponding linear response theory (Sect. 2.2.3) begins by considering it to be bandlimited.

To create the connectivity matrix *W*, each neuron *j* from population *b* is randomly connected (without replacement) to $p_{ab} N_{a}$ neurons from population *a*, resulting in a fixed out-degree network. From these possible synapses, each one has a probability of $\rho _{ab}^k$ of being ensheathed with strength $s_{en}^k$. The parameters $\rho _{ab}^k$ and $s_{en}^k$ vary throughout this work, with their values clearly indicated within the figures.

#### Average Population Firing Rates Derived from Self-Consistency Relationship

We define the average firing rate of neuron *i* as $r_i = \langle y_i(t) \rangle$ and the corresponding average population firing rate as

$$\begin{aligned} {\hat{r}}_{a} = \frac{1}{N_{a}}\sum _{i \in a} \langle y_i(t) \rangle . \end{aligned}$$

To solve for the steady state firing rates, we begin by noting that if the effective mean input into neuron *i*, $\mu _i^\text {eff}$, and the effective variance of these inputs, $\left( \sigma _i^\text {eff}\right) ^2$, is known, then the membrane potential for each neuron evolves according to the stochastic different equation

7 $$\begin{aligned} \tau _m \frac{dV_i}{dt} = -\left( V_i - \mu _i^\text {eff}\right) + \psi (V_i) + \sigma _i^\text {eff} \xi _i(t). \end{aligned}$$

One can solve for the steady state firing rates by writing down the corresponding Fokker–Plank equation and solving for flux through the threshold potential, $V_{th}$ (Richardson 2008). However, in our case, the effective mean input and variance have the form

$$\begin{aligned} \mu _i^\text {eff}&= E_L + \mu _{i,bg} + \mu _{i,\text {ffwd}} + \sum _b \sum _{j\in b} \left( \int ^\infty _{-\infty } W_{ij}J_{ij}(t) dt\right) r_j, \end{aligned}$$

and

$$\begin{aligned} \left( \sigma _i^\text {eff}\right) ^2&= 2\tau _m\cdot \left( \sigma _{i,bg}^2 + \sigma _{i,\text {ffwd}}^2 + \sigma _{gl}^2+\sum _b \sum _{j\in b} \left( \int ^\infty _{-\infty } W_{ij}J_{ij}(t) dt\right) ^2r_j\right) , \end{aligned}$$

both of which depend on the firing rates $r_j$. The form of these dependencies follows from the same diffusion approximation and Poisson spike train assumption mentioned previously, and follows closely with similar work (Trousdale et al. 2012; Veit et al. 2023). As a result, our problem must satisfy the self-consistency relationship

$$\begin{aligned} r_i = r_i(\mu ^\text {eff}_i,\sigma _i^\text {eff}). \end{aligned}$$

This relationship can be satisfied by repeating the process detailed above via fixed point iteration, updating both the steady state firing rates, as well as $\mu _i^\text {eff}$ and $\sigma _i^\text {eff}$.

While analytical results have been found for steady state firing rates for linear and uncoupled neurons (e.g., see Fourcaud and Brunel (2002) for an integrate-and-fire neurons), this work relies on numerical methods developed in Richardson (2007, 2008) for our non-linear integrate-and-fire network. This numerical scheme requires the restriction of the voltage to be above a lower bound, $V_{lb}$, but it is chosen to be sufficiently negative so that its precise value has a negligible impact on the evaluation of the firing rate.

With these quantities in hand, we can also numerically estimate the linear response function, ${\tilde{A}}_i(f)$, and the power spectrum of the baseline spike trains, ${\tilde{C}}_{ii}^0(f)$, of each neuron. In short, this method begins by considering Eq. 7, and then applying a weak perturbation to the membrane potential to the corresponding Fokker–Planck equation. After approximating the solution to first order, one can derive both the linear response function and the spike-train power spectrum.

We now seek to simplify this *N*-coupled system of self-consistency relationships significantly by deriving the corresponding relationship for the average firing rate across the populations, ${\hat{r}}_a$. We begin by noting that the leak reversal potential, as well as the mean and variance of external inputs, are the same for all neurons in population *a*. Thus, the average effective input $\hat{\mu }^\text {eff}_a$ into population *a* can be written as

$$\begin{aligned} \hat{\mu }_a^\text {eff}&= \frac{1}{N_a}\sum _{i\in a} \mu _i^\text {eff} \\&= E_L + \mu _{a,bg} + \mu _{a,\text {ffwd}} + \frac{1}{N_a}\sum _{i\in a}\sum _{b} \sum _{j\in b} W_{ij}r_j, \end{aligned}$$

where we have also used the fact that the the integral of the synaptic interaction is 1 by design, even in the presence of glial ensheathment. We can approximate the last term in this sum as follows

$$\begin{aligned} \frac{1}{N_a}\sum _{i\in a}\sum _{b}\left[ \sum _{j\in b} W_{ij}r_j\right]&\approx \frac{1}{N_a}\sum _b\left[ \sum _k p_{ab}N_{a}w_{ab}\left( 1- s_{en}^k\right) \rho _{ab}^k\sum _{j\in b} r_j\right] , \\&= \frac{1}{N_a}\sum _b\left[ p_{ab}N_{a}w_{ab}\left( 1- \sum _k s_{en}^k\rho _{ab}^k\right) \sum _{j\in b} r_j\right] , \\&= \sum _b\left[ p_{ab}w_{ab}\left( 1- \sum _k s_{en}^k\rho _{ab}^k\right) N_b \cdot {\hat{r}}_b\right] , \\&= \sum _b\left( M_{ab}\left( 1-{\hat{s}}_{en}^{ab}\right) \right) {\hat{r}}_b, \end{aligned}$$

where

8 $$\begin{aligned} {\hat{s}}_{en}^{ab} = \sum _ k s_{en}^k\rho _{ab}^k, \end{aligned}$$

is the weighted average of the ensheathment strength from population *b* to *a*, and

$$\begin{aligned} M_{ab} = p_{ab}N_{b}w_{ab}, \end{aligned}$$

is the effective connectivity strength from population *b* to *a* in the absence of glial ensheathment. The first line follows from the fact that a neuron in population *b* makes on average $p_{ab}N_a\rho _{ab}^k$ connections with an ensheathment strength of $s_{en}^k$ to a neuron in population *a*.

The calculation for the average variance proceeds in a similar fashion,

$$\begin{aligned} \left( \hat{\sigma }_i^\text {eff}\right) ^2&= \frac{1}{N_a} \sum _{i\in a} \left( \sigma _i^\text {eff}\right) ^2 \\&= \left( \sigma _{i,bg}^2 + \sigma _{i,\text {ffwd}}^2 + \sigma _{gl}^2+\frac{1}{N_a}\sum _{i\in a}\sum _b \sum _{j\in b} \frac{W_{ij}^2}{4\tau _s^{ij}}r_j\right) \cdot 2\tau _m, \end{aligned}$$

where we have calculated the integral of the square of the synaptic interaction term. The last term in the parentheses can be approximated similar to the steps above to find

$$\begin{aligned} \frac{1}{N_a}\sum _{i\in a}\sum _b \sum _{j\in b} \frac{W_{ij}^2}{4\tau _s^{ij}}r_j&\approx \frac{1}{N_a}\sum _b\left[ \sum _kp_{ab}N_a\frac{\left( w_{ab}(1-s_{en}^k)\right) ^2}{4\tau _s\left( 1-\beta s_{en}^k\right) }\rho _{ab}^k \sum _{j\in b} r_j\right] \\&= \frac{1}{N_a}\sum _b\left[ \frac{p_{ab}N_aw_{ab}^2}{4\tau _s}\left( \sum _k\frac{(1-s_{en}^k)^2}{1-\beta s_{en}^k}\rho _{ab}^k\right) \sum _{j\in b} r_j\right] \\&= \sum _b \frac{M_{ab}w_{ab}}{4\tau _s}\cdot \gamma _{s_{en}}^{ab}\cdot {\hat{r}}_b, \end{aligned}$$

where

$$\begin{aligned} \gamma _{s_{en}}^{ab} = \left( \sum _k\frac{(1-s_{en}^k)^2}{1-\beta s_{en}^k}\rho _{ab}^k\right) , \end{aligned}$$

captures the effect ensheathment has on this higher-order correction term.

These calculations yield a self-consistency relationship for the average population firing rates

9 $$\begin{aligned} {\hat{r}}_a = {\hat{r}}_a(\hat{\mu }^\text {eff}_a,\hat{\sigma }_a^\text {eff}), \end{aligned}$$

where

$$\begin{aligned} \hat{\mu }_a^\text {eff}&= E_L + \mu _{a,bg} + \mu _{a,\text {ffwd}} + \sum _b M_{ab}\left( 1-{\hat{s}}_{en}^{ab}\right) r_b, \end{aligned}$$

and

$$\begin{aligned} \left( \hat{\sigma }_a^\text {eff}\right) ^2&= \left( \sigma _{a,bg}^2 + \sigma _{a,\text {ffwd}}^2 + \sigma _{gl}^2+\sum _b \frac{M_{ab}w_{ab}}{4\tau _s}\cdot \gamma _{s_{en}}^{ab}\cdot {\hat{r}}_b\right) \cdot 2\tau _m. \end{aligned}$$

This result is written to explicitly highlight the dependence on ensheathment strength. When there is no ensheathment (i.e., $s_{en}^{ab} =0$ and $\gamma _{s_{en}}^{ab} = 1$), this self-consistency relationship simplifies to rely only on terms involving the traditional effective connectivity strength, as derived in previous mean-field analyses (e.g., Rosenbaum et al. (2017) and Bos et al. (2020)). Similar to before, we can now craft a corresponding stochastic differential equation, namely

10 $$\begin{aligned} \tau _m \frac{dV_a}{dt} = -\left( V_a - \hat{\mu }_a^\text {eff}\right) + \psi (V_a) + \hat{\sigma }_a^\text {eff} \xi _a(t), \end{aligned}$$

which can be used to numerically find both linear response function, ${\tilde{A}}_a(f)$, and the power spectrum of the baseline spike trains, ${\tilde{C}}_{aa}^0(f)$, for each neuronal population following the same steps as before (i.e., following the algorithm outlined in Richardson (2007, 2008)).

#### Average Power- and Cross-Spectrums Derived from Linear Response Theory

We now turn our attention to higher-ordered network statistics. Specifically, we aim describe correlations across population spike trains via a formula for the power and cross-spectrum matrix. The cross-correlation function of neurons *i* and *j* at lag *h* is defined to be $C_{ij}(h) = \langle (y_i(t) - r_i)(y_j(t+h) - r_j)\rangle$, where $\langle \cdot \rangle$ denotes the average across time, typically taken to be over the length of an entire trial. The corresponding average population auto- and cross-covariance functions are then defined as

$$\begin{aligned} {\hat{C}}_{a b}(h)&= \frac{1}{N_{a}}\frac{1}{N_{b}} \sum _{i \in a}\sum _{j \in b} C_{ij}(h). \end{aligned}$$

The Fourier transform of these functions then yields the corresponding power spectrum, $\hat{{\tilde{C}}}_{aa}(f)$, and cross-spectrum, $\hat{{\tilde{C}}}_{ab}(f)$.

To derive a formula for $\hat{{\tilde{C}}}(f)$, we follow previous work (Doiron et al. 2004; Lindner et al. 2005; Trousdale et al. 2012; Veit et al. 2023) and linearize each neuron’s spike train around a realization of the spiking output in the absence of recurrent connections, $y_i^0(t)$. We begin by explicitly assuming that the synaptic connections, $W_{ij}$, are weak and that the global noise process is band-limited. This allows us to approximate the spike response from neuron *i* in the Fourier domain as

$$\begin{aligned} {\tilde{y}}_i(f) = {\tilde{y}}_i^0(f) + {\tilde{A}}_i(f)\left[ \sigma _{gl}\sqrt{2\tau _m}\tilde{\eta }_{gl}(f) + \sum _b\sum _{j\in b} W_{ij}{\tilde{J}}_{ij}(f){\tilde{y}}_j(f)\right] \end{aligned}$$

where ${\tilde{y}}_i(f)$ is the zero-mean Fourier transform of the spike train, $\tilde{\cdot }$ denotes the Fourier transform of the other quantities, and ${\tilde{A}}_i(f)$ is the linear response of the postsynaptic neuron derived in the previous section (Gardiner 2009). Averaging the spike response equation across population *a* then yields

$$\begin{aligned} \frac{1}{N_a} \sum _{i\in a} {\tilde{y}}_i(f)&= \frac{1}{N_a} \sum _{i\in a} \left[ {\tilde{y}}_i^0(f) + \sigma _{gl}\sqrt{2\tau _m}\tilde{\eta }_{gl}(f){\tilde{A}}_i(f) \right. \\&\quad \left. + {\tilde{A}}_i(f)\sum _b\sum _{j\in b} W_{ij}{\tilde{J}}_{ij}(f){\tilde{y}}_j(f)\right] , \\&\approx \frac{1}{N_a} \sum _{i\in a} {\tilde{y}}_i^0(f) + \sigma _{gl}\sqrt{2\tau _m}\tilde{\eta }_{gl}(f){\tilde{A}}_a(f) \\&\quad + \frac{{\tilde{A}}_a(f)}{N_a} \sum _{i\in a}\sum _b \left[ \sum _{j\in b} W_{ij}{\tilde{J}}_{ij}(f){\tilde{y}}_j(f)\right] , \end{aligned}$$

where we have made the approximation ${\tilde{A}}_a(f) \approx {\tilde{A}}_i(f)$. The third term in this sum can be simplified following the same reasoning as in Sect. 2.2.2,

11 $$\begin{aligned} \frac{{\tilde{A}}_a(f)}{N_a} \sum _{i\in a} \sum _{b}&\left[ \sum _{j\in b} W_{ij}{\tilde{J}}_{ij}(f){\tilde{y}}_j(f)\right] \nonumber \\&\,\,\,\,\approx \frac{{\tilde{A}}_a(f)}{N_a}\sum _{b} \left[ \sum _k p_{ab} N_a w_{ab}(1-s_{en}^k){\tilde{J}}_{ab}^k(f)\rho _{ab}\sum _{j\in b}{\tilde{y}}_j(f)\right] \nonumber \\&\,\,\,\,= \frac{{\tilde{A}}_a(f)}{N_a}\sum _{b}\left[ p_{ab} N_a w_{ab} \sum _k (1-s_{en}^k){\tilde{J}}_{ab}^k(f)\rho _{ab}^k \sum _{j\in b}{\tilde{y}}_j(f)\right] \nonumber \\&\,\,\,\,= {\tilde{A}}_a(f) \sum _{b}M_{ab} \hat{{\tilde{J}}}_{ab}(f) \hat{{\tilde{y}}}_b(f), \end{aligned}$$

where

$$\begin{aligned} \hat{{\tilde{y}}}_b(f) = \frac{1}{N_b}\sum _{j\in b}{\tilde{y}}_j(f) \end{aligned}$$

denotes the Fourier transform of the concatenated spike trains from each population,

$$\begin{aligned} {\hat{y}}_b(t) = \frac{1}{N_b}\sum _{j\in b}{y}_j(t), \end{aligned}$$

and

12 $$\begin{aligned} \hat{{\tilde{J}}}_{ab}(f) = \sum _k (1-s_{en}^k){\tilde{J}}_{ab}^k(f)\rho _{ab}^k \end{aligned}$$

is a modified synaptic kernel that accounts for ensheathment strength. We note that in the case of a fixed in-degree network, ${\tilde{A}}_a(f) = {\tilde{A}}_i(f)$ (i.e., all neurons have the same linear response function). Here, we consider a fixed out-degree network, which has the benefit of ensuring that a neuron in population *b* makes exactly $p_{ab}N_a$ connections to a neuron in population *a*, which appears in the derivation of Eq. 11.

Using this simplified form, we can now write

13 $$\begin{aligned} \hat{{\tilde{y}}}_a(f) = \hat{{\tilde{y}}}_a^0(f) + {\tilde{A}}_a(f)\left[ \sigma _{gl}\sqrt{2\tau _m}\tilde{\eta }_{gl}(f) + \sum _b M_{ab}\hat{{\tilde{J}}}_{ab}(f)\hat{{\tilde{y}}}_b(f)\right] , \end{aligned}$$

for $a = 1,...,P$. It is important to note that, unlike the final self-consistency relationship used to estimate average firing rates in Sect. 2.2.2, the effect of ensheathment strength here is incorporated into the modified synaptic kernel. As a result of this derivation, we have *P* linear equations, which we can solve for the *P* unknowns $\hat{{\tilde{y}}}_a(f)$. We denote the solution to this linear system as $\varvec{\hat{{\tilde{y}}}}(f)$. This is a $P \times 1$ vector that can be used to find the averaged power spectrum and cross-spectrum matrix, which is a $P \times P$ matrix denoted as $\varvec{\hat{{\tilde{C}}}}(f)$. Aside from the use of our modified synaptic kernel, this calculation follows exactly as in Veit et al. (2023), and results in the following expression

$$\begin{aligned} \varvec{\hat{{\tilde{C}}}}(f)&= \langle \varvec{\hat{{\tilde{y}}}}(f)\varvec{\hat{{\tilde{y}}}^*}(f)\rangle \\&= (\varvec{I}-\varvec{K}(f))^{-1}\left[ \varvec{\hat{{\tilde{C}}}^0}(f) +\sigma _{gl}^2 2\tau _m\langle \tilde{\eta }_{gl}(f)\tilde{\eta }_{gl}^*(f)\rangle \varvec{\hat{{\tilde{A}}}}\textbf{1}\varvec{\hat{{\tilde{A}}}}\right] (\varvec{I}-\varvec{K^*}(f))^{-1}, \end{aligned}$$

with

$$\begin{aligned} \varvec{K}(f) = \varvec{\hat{{\tilde{A}}}}(f)(\varvec{M}\odot \varvec{\hat{{\tilde{J}}}}(f)), \end{aligned}$$

where $\odot$ denotes element wise-multiplication, $\varvec{\hat{{\tilde{A}}}}(f)$ is a diagonal matrix of linear response functions, $\varvec{M}$ is the matrix of effective connectivity strengths, $\varvec{\hat{{\tilde{J}}}}(f)$ is the matrix of modified synaptic kernels, $\textbf{1}$ is the matrix of all ones, and $\varvec{\hat{{\tilde{C}}}^0}(f)$ is a diagonal matrix with entries

$$\begin{aligned} \frac{1}{N_a}{\tilde{C}}_{aa}^0(f) = \frac{1}{N_a}\langle {\tilde{y}}_a^0(f){\tilde{y}}_a^0(f)\rangle . \end{aligned}$$

Similarly, following the work of Lindner et al. (2005) we can extend this to account for global noise that takes the form of Gaussian white noise (with infinite variance) and find

14 $$\begin{aligned} \varvec{\hat{{\tilde{C}}}}(f)&= \langle \varvec{\hat{{\tilde{y}}}}(f)\varvec{\hat{{\tilde{y}}}^*}(f)\rangle \nonumber \\&= (\varvec{I}-\varvec{K}(f))^{-1}\left[ \varvec{\hat{{\tilde{C}}}^0}(f) +\sigma _{gl}^2 2\tau _m\langle \tilde{\eta }_{gl}(f)\tilde{\eta }_{gl}^*(f)\rangle \varvec{\hat{{\tilde{A}}}}\textbf{1}\varvec{\hat{{\tilde{A}}}} - \sigma ^2_{gl}2\tau _m\left| \varvec{\hat{{\tilde{A}}}_N}(f)\right| ^2\right] \end{aligned}$$

15 $$\begin{aligned}&\,\,\,\, \cdot (\varvec{I}-\varvec{K^*}(f))^{-1}, \end{aligned}$$

where $\varvec{\hat{{\tilde{A}}}_N}(f)$ is a diagonal matrix with entries ${\tilde{A}}_a(f)/N_a$. In this work, we consider global noise with a magnitude small relative to the independent noise, leading to this correction term having a minimal effect, as it is orders of magnitude smaller than the other terms within the brackets.

#### Comparison to Naïve Mean-Field Approach

It is worth noting that our self-consistency relationship (Eq. 9), as well the average spike response from a neuron (Eq. 13), vary from what one would find from a more naïve mean-field approach. Specifically, one could first average across the network to find the average level of ensheathment experienced across the populations (Eq. 8), and then update the synaptic weights and time constants to find

$$\begin{aligned} {\overline{W}}_{ab}&= w_{ab}\cdot (1-{\hat{s}}_{ab}), \\ {\bar{J}}_{ab}(t)&= \frac{t-\tau _{\text {delay},j}}{(\tau _s\cdot (1-{\hat{s}}_{ab}))^2}\cdot \exp \left[ -\frac{t-\tau _{\text {delay},j}}{\tau _s\cdot (1-{\hat{s}}_{ab})}\right] {\mathcal {H}}(t-\tau _{\text {delay},j}). \end{aligned}$$

It is clear that these terms do not appear in our derivations, indicating that this naïve approach would have noticeable deviations in its approximations of the average firing rates, power spectrum and cross-spectrum.

### Default Network Connectivity and Baseline Dynamics for Network Without Glial Ensheathment

#### Default Connectivity

**Fig. 3:**
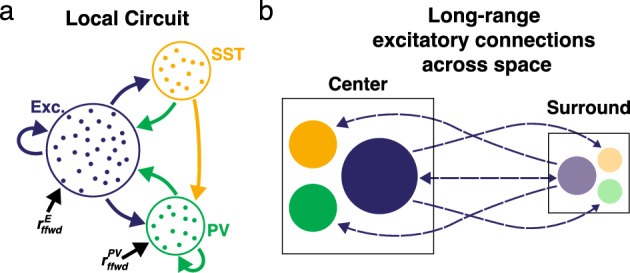
**a** Network schematic showing the local connectivity rules across the neuronal populations (blue: excitatory, green: PV, and yellow: SST). **b** Network schematic showing the long range excitatory connections between the ‘center’ (solid colors) and ‘surround’ (translucent colors) locations (Color figure online)

The theory developed in Sect. 2.2 holds for arbitrary number of neuronal populations and various network configurations. To demonstrate its utility, for the rest of this work we will consider a model of mouse V1 adapted from Veit et al. (2023). The model consists of three neuronal subtypes, *e* (excitatory), *p* (PV), and *s* (SST), spread across two discrete retinotopic locations: a ‘center’ and a ‘surround’. For neurons located in the same retinotopic space, the probability of connecting across and within neuron subclasses follows from previous experimental and modeling works (Pfeffer et al. 2013; Litwin-Kumar et al. 2016; Bos et al. 2020; Veit et al. 2023). As illustrated in Fig. 3a, within this local circuit, there are many direct connections both across and within the excitatory, PV, and SST subpopulations. However, we highlight that a distinguishing feature of SST neurons is that they neither directly inhibit other SST neurons nor receive direct inhibition from PV neurons. Following the work of Keller et al. (2020), only the excitatory population has long-range, outgoing connections across spatial locations (Fig. 3b). The probability of these long-range connections differs depending on the postsynaptic population at the other location. These are chosen to qualitatively align with the parameters used in Keller et al. (2020), which fitted a rate-based model to experimental data collected from L2/3 of a mouse presented with different visual stimuli. All connection probability parameters are listed in Table 2.

**Table 2:** Probability of connections $p_{ab}$ (from population *b* to *a*; columns are the presynaptic populations, and the rows are the postsynaptic)

	Same spatial location			Diff. Spat. Loc
	Exc	PV	SST	Exc
Exc	0.07	0.15	0.10	0.02
PV	0.05	0.10	0.10	0.03
SST	0.10	0.00	0.00	0.08

Parameters values are the same as Veit et al. (2023)

While our theory allows for the probability of ensheathment to depend on pre- and post-synaptic partners, for the remainder of this work, we make the simplifying assumption that these values depend only on the pre-synaptic population and remain constant across spatial locations. Under this simplification, we express $\rho _b^k$ as the probability of a synapse from neuronal subclass *b* being ensheathed with strength $s^k_{en}$.

#### Numerical Details

All remaining parameter values are the same as in Veit et al. (2023) and can be found in Table 3. These parameter choices are consistent with other modeling and experimental work that investigate mouse V1 (Pfeffer et al. 2013; Litwin-Kumar et al. 2016; Bos et al. 2020; Keller et al. 2020). We also note that the third large interneuron class, VIP, is implicitly accounted for as a fixed negative background current into SST neurons. This follows the work from Veit et al. (2023), where VIP neurons are accounted for as simply a feedforward source of inhibition onto SST cells.

The spiking simulations were completed with Euler’s method using a time step of 0.025 msec for a total of $1\times 10^6$ msec of simulation time. Unless otherwise denoted, we will report the statistics (e.g., firing rate, power spectrum of spike train) from the center location.

To numerically estimate the auto- and cross-correlation functions from our numerical simulations, we first start by representing the spike trains of individual neurons as a binary sequence:

$$\begin{aligned} y_i(t) = {\left\{ \begin{array}{ll} 1 & \text {if neuron i fired during the interval } (t, t+\Delta t)\\ 0 & \text {otherwise} \end{array}\right. }, \end{aligned}$$

where $\Delta t = 0.001$ seconds. We then sum these sequences across subpopulations in different spatial locations, after which we apply MATLAB’s built-in xcorr() function with a maximum window length of 0.25 s to yield the numerical estimation (MATLAB 2023). We can then take the Fourier transform of these functions to find the power spectrum, $\hat{{\tilde{C}}}_{aa}(f)$, and cross-spectrum, $\hat{{\tilde{C}}}_{ab}(f)$. We will use these quantities to also estimate the coherence across spatial locations, which is defined as

16 $$\begin{aligned} \text {coherence}(f) = \frac{\left| \hat{{\tilde{C}}}_{ab}(f)\right| ^2}{\hat{{\tilde{C}}}_{aa}(f) \cdot \hat{{\tilde{C}}}_{bb}(f)}. \end{aligned}$$

This coherence measure is a normalized measure of synchrony, with values ranging from 0 (no synchrony) to 1 (high synchrony).

**Table 3:** Default neuronal and network parameters

Parameter	Value	Description
$N_{e}$	4000	Number of excitatory neurons at each location
$N_{p}$	500	Number of PV neurons at each location
$N_{s}$	500	Number of SST neurons at each location
$E_l$	$-60$ mV	Resting potential
$V_{th}$	20 mV	Threshold potential
$V_{re}$	$-75$ mV	Reset potential
$\tau _m$	5.4 msec	Membrane time constant
$\tau _s$	0.6 msec	Synaptic time constant
$\tau _\text {ref}$	1.2 msec	refractory period
$\Delta _T$	$-50$ mV	Exponential shape parameter (soft threshold)
$V_{lb}$	$-100$ mV	Lower bound for voltage
$\tau _\text {delay}$	1.8 msec	Synaptic delay
$w=w_{\alpha e}$	0.48 mV msec	Synaptic strength of excitatory connections
*g*	4	Amplification of inhibitory connection strength
$w_{ap}=w_{as}$	$-gw$	Synaptic strength of inhibitory connections
$[\mu _{e,bg},\mu _{p,bg},\mu _{s,bg}]$	$[3,3,-4.52]$ mV	Background mean
$[\sigma ^2_{e,bg},\sigma ^2_{p,bg},\sigma ^2_{s,bg}]$	[2.12, 2.12, 6.77] mV	Background standard deviation
$[\mu _{e,\text {ffwd}},\mu _{p,\text {ffwd}},\mu _{s,\text {ffwd}}]$	[2.25, 2.25, 0] mV	Feedforward mean
$[\sigma ^2_{e,\text {ffwd}},\sigma ^2_{p,\text {ffwd}},\sigma ^2_{s,\text {ffwd}}]$	[1.84, 1.84, 0] mV	Feedforward standard deviation
$\sigma _{gl}$	0.25 mV	Global noise standard deviation
$1-\beta$	0.4	$\tau _s^\text {min}/\tau _s$

All parameters values, except for $1-\beta$ (see Sect. 2.1.3), are the same as Veit et al. (2023). Changes to any parameter are indicated in the figure caption

#### Default Network Dynamics

**Fig. 4:**
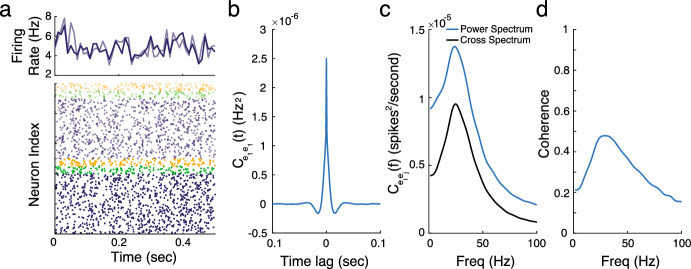
**a** (bottom) Raster plot indicating the spike times of a sample of neurons (from the excitatory (blue), PV (green) and SST (yellow) populations at the ‘center’ (solid) and ‘surround’ (translucent), (top) Average firing rate (averaged over 0.01 s time windows) for the excitatory neurons in the two spatial locations. **b** Auto-covariance function for the excitatory population for different time lags. **c** The power spectrum for the excitatory population (blue) and cross-spectrum between excitatory populations at different spatial locations (black) across different frequencies. **d** Coherence across frequencies between the two excitatory populations (Color figure online)

For the default parameter set without any glial ensheathment, we observed that the neurons in the network are spiking asynchronously, with low correlations within and across populations, as illustrated in the raster plot shown in Fig. 4a (bottom). Figure 4a (top) shows the time courses of the average population firing rates for the two excitatory populations, calculated using a sliding 0.01 s time window.

Figure 4b–d shows the estimated auto-correlation, power, cross-spectrum, and coherence for the default network. For these parameter values, the network exhibits a strong gamma rhythm, evident by the bump in the spectrum occurring in the 20–50 Hz range, with moderate levels of coherence at these frequencies. We define the “gamma power” as the peak of the power spectrum within this frequency range, and the “gamma frequency” as the frequency at which this peak occurs. Additionally, we define “gamma coherence” as the coherence value at the corresponding gamma frequency.

## Modeling Results

### Replication of Experimental Observations

**Fig. 5:**
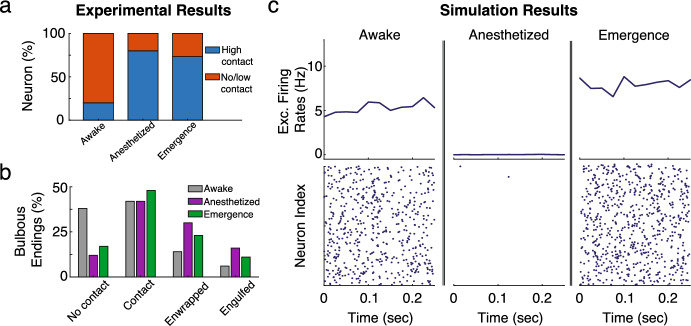
**a** Modified panel from Haruwaka et al. (2024) showing that the number of neurons with multiple synapses with nearby microglia increases significantly after the administration of isoflurane, and remains after its removal (i.e., ‘emergence’). **b** Modified panel from Haruwaka et al. (2024) showing how the coverage of GABAergic synapses change between the three states. **c** Simulated raster plots (bottom) and average firing rates (top), averaged over 0.025 s time windows, across the three experimental states using ensheathment parameters found in Table 4. In addition, for the anesthetized state, the mean and variance of the feedforward input was set to $[\mu _{e,\text {ffwd}},\mu _{p,\text {ffwd}},\mu _{s,\text {ffwd}}] = [0, 0, 0]$ mV, and $[\sigma ^2_{e,\text {ffwd}},\sigma ^2_{p,\text {ffwd}},\sigma ^2_{s,\text {ffwd}}] =[0, 0, 0]$ mV (Color figure online)

With the spiking network and mean-field theory established, we first aim to replicate the recent results of Haruwaka et al. (2024). In that study, they observed that during emergence from anesthesia induced by the administration of isoflurane, neurons in layer 2/3 of the cortex became hyperactive, with a significant increase in firing rates. To explain these results, they tracked changes in microglia-neuronal interactions, specifically focusing on the number and strength of the connections between these two cell types. They found that the percentage of neurons with nearby microglia increased from 20% to nearly 80% from the awake to the anesthetized and post-anesthetized (i.e., “emergence”) states (Fig. 5a). Interestingly, they also found that these interactions predominantly affected GABAergic synapses (i.e., inhibitory inputs). Using confocal microscopy, they classified these interactions into four subtypes based on surface contact: no contact, less than 50% surface coverage (low contact), more than 50% surface coverage (enwrapped), and complete engulfment. These levels also changed significantly across the three states, with an increase in enwrapped and engulfed synapses during and after isoflurane administration (Fig. 5b). Finally, they confirmed through super-resolution and electron microscopy that the tips of microglial bulbous endings shielded PV pre-synaptic terminals by protruding into the cleft, consistent with our microscale model.

With these experimental findings as a reference, we ask whether our spiking model can replicate the observed increase in hyperexcitability from the awake to emergence state via changes in glial ensheathment alone. We consider four levels of ensheathment strength: $s_{en}^1 = 0$ (no contact), $s_{en}^2=0.33$ (contact), $s_{en}^3=0.67$ (enwrapped), and $s_{en}^4=1$ (engulfed). The probability of ensheathment for out-going inhibitory synapses (both PV and SST) was taken from the experimental data and can be found in Table 4. Meanwhile, all other synapses were placed in the no contact state. In addition to changing the ensheathment levels, the anesthetized state, which exhibits little to no spiking due to the inhibition of external driving forces, was modeled by removing the feedforward input. Importantly, our analysis focuses only on the steady state behavior in these three distinct states, and not on the transient behavior that occurs as the system transitions between them.

**Table 4:** Probability of ensheathment for all outgoing inhibitory connections, $\rho _{p}$ and $\rho _{s}$, as used in Fig. 5

	No contact	Contact	Enwrapped	Engulfed
	($s_{en}^1 = 0$)	($s_{en}^2 = 0.33$)	($s_{en}^3 = 0.67$)	($s_{en}^4 = 1$)
Awake	0.800	0.136	0.045	0.019
Anesthetized	0.200	0.382	0.273	0.145
Emergence	0.267	0.433	0.203	0.097

**Fig. 6:**
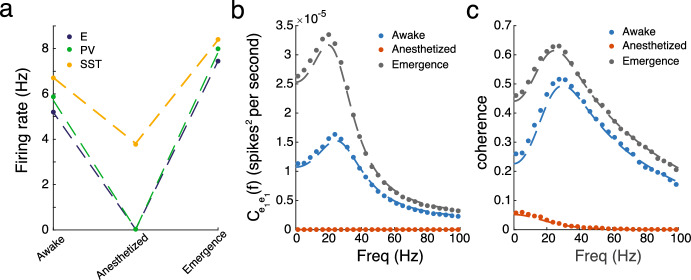
Results of the spiking model (dots) and mean-field approximation (dashed-line) for the **a** firing rates, **b** power spectrum, and **c** coherence across the awake, anesthetized and emergence states using the ensheathment parameters found in Table 4. In addition, for the anesthetized state the mean and variance of the feedforward input was set to $[\mu _{e,\text {ffwd}},\mu _{p,\text {ffwd}},\mu _{s,\text {ffwd}}] = [0, 0, 0]$ mV, and $[\sigma ^2_{e,\text {ffwd}},\sigma ^2_{p,\text {ffwd}},\sigma ^2_{s,\text {ffwd}}] =[0, 0, 0]$ mV (Color figure online)

Figure 5c illustrates the results of the model with raster plots (bottom) and the average population firing rates for the excitatory population (top), calculated using a sliding 0.025 s time window, for the three different states. As expected, there is a significant drop in excitatory firing rates from the awake to the anesthetized state, which is not due to changes in glial ensheathment but rather the removal of feedforward inputs. Once the model enters the emergence state, where glial ensheathment levels remain elevated and the feedforward inputs are restored, the network enters a hyperactive state, showing a 43% increase in excitatory firing rates. These baseline results demonstrate that the “effective” glial ensheathment model derived in Sect. 2.1 can successfully replicate this key experimental result when implemented into a large-scale spiking model. We also report significant increases in firing rates across the PV and SST populations (Fig. 6a).

It is worth noting that, although we have experimental data regarding the probability of a synapse being ensheathed at a certain level (e.g., ‘contact’, ‘enwrapped’), as outlined in Table 4, it remains an open question how these classification levels map onto the strength of the ensheathment parameter $s_{en}$, and how variable the geometry of this ensheathment is from synapse to synapse (i.e., symmetric vs. non-symmetric). Ideally, we would have precise measurements of protrusion depth for each of these classification levels, which would require super-resolution imaging at the scale of microns. We could then map these measurements onto $s_{en}$ directly using the methods outlined in Sect. 2.1. In the absence of this data, our microscale investigation offers some insight. Namely, due to the linear relationship between glial protrusion and synaptic parameters (Fig. 2), the behavior observed here is robust to these parameter choices. Additionally, the model predicts that variations in glial protrusion geometry, regardless of symmetry, play a minimal role and do not need to be precisely classified experimentally.

### Higher Ordered Statistics and Verification of Linear Response Theory

The implications of this hyperactivity on cortical processes that depend on higher-order network statistics remain an open question. Here, we focus on network dynamics in V1, examining the strength and synchrony of gamma rhythms, which are thought to play a role in spatially integrating visual information. Following the steps in Sect. 2.3, we calculate the average power spectrum and cross-spectrum for the excitatory populations at both spatial locations. Figure 6b shows a clear increase in the power spectrum across all frequencies, with the largest increase occurring within the gamma frequency range. Similarly, Fig. 6c shows a significant increase in the coherence (i.e., a normalized measure of synchrony; see Eq. 16) of these gamma oscillations. Thus, in addition to shaping the firing rates of the network, our model predicts that glial ensheathment can lead to significant changes in synchronous activity. This is also a testable prediction. Veit et al. (2017, 2023) examined coherence across V1 in head-fixed mice viewing oriented gratings in the context of optogenetic stimulation of different interneuron subclasses using two laminar multielectrode arrays to estimate the local field potential at two distinct locations. It would be interesting to replicate those experiments as the animal emerges from an anesthetized state following a similar protocol as Haruwaka et al. (2024) to see if these increases in gamma power are observed.

With this parameter set and results in mind, we now turn to the linear response theory and the corresponding mean-field approximation derived in Sect. 2.2 to determine if similar results hold. We find impressive agreement for the firing rates, power spectrum, and coherence in both the awake and emergence states (Fig. 6). Recall that the heterogeneity induced by glial ensheathment considered here results in synaptic strengths and interaction kernels being drawn from entirely distinct distributions. This is a significant departure from many previous studies that consider heterogeneous connection strengths distributed according to a normal distribution, ${\mathcal {N}}(J, \sigma )$ (Rajan and Abbott 2006; Rajan et al. 2010; Mastrogiuseppe and Ostojic 2018). Furthermore, this goes beyond the binary case (where each synapse was either ensheathed or unsheathed) considered in the spiking simulations in Handy and Borisyuk (2023), not only by considering multiple strengths at once but also through the development of this mean-field approximation. Lastly, this mean-field approximation runs at a fraction of the computational cost of spiking simulations (9 s vs. 4.3 h on a personal laptop), the latter of which require long trials for accurate estimation of the power spectrum and cross-spectrum.

### Investigating the Impact of Glial Ensheathment PV Versus SST Interneurons

We now utilize the mean-field approximation to perform a broader parameter sweep and disentangle the effects of ensheathing both PV and SST interneurons. Specifically, we will consider networks with *either* PV or SST interneuron ensheathment across a range of ensheathment probabilities and strengths. To simplify this exploration, we consider only binary levels of ensheathment, where every synapse from population *b* is either ensheathed with strength $s_{en}$ with probability $\rho _b$ or unsheathed with probability $1-\rho _b$.

**Fig. 7:**
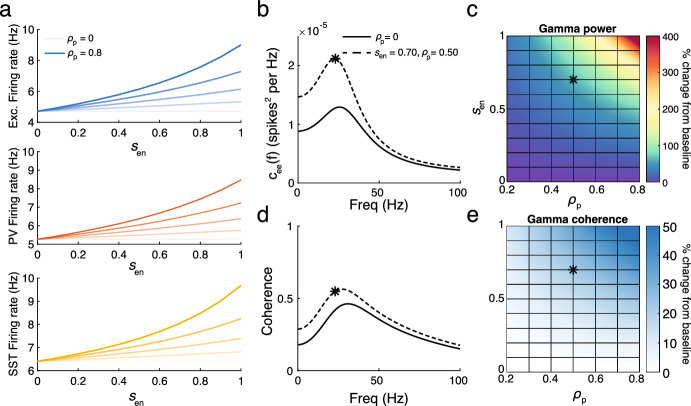
**a** Firing rates of excitatory (top), PV (middle) and SST (bottom) neurons for a range of ensheathment strengths and probability of ensheathment of PV synapses ($\rho _{p} = 0, 0.2, 0.4, 0.6, 0.8$ from light to dark shades). **b** Power spectrum for the excitatory population for $\rho _{p} = 0$ (solid) and 0.5 (dashed) for $s_{en} = 0.7$. The star indicates the gamma power. **c** The percentage change in gamma power from baseline ($\rho _{p}=0$) for a range of ensheathment strengths and probabilities $\rho _{p}$. The star indicates the point in parameter space used in panel **b**. **d** The same as panel b but for coherence. **e** The same as panel **c**, but for gamma coherence (Color figure online)

Figure 7 shows the results for the ensheathment of PV neurons. We find that across a range of probabilities and strengths, this reliably leads to an increase in firing rates across the three populations, with larger deviations from baseline occurring for greater values of $s_{en}$ and $\rho _p$ (Fig. 7a). Furthermore, we observe increases in the power spectrum of the excitatory population over a range of lower frequencies, along with a consistent increase in gamma power (Fig. 7b, c). The coherence, specifically the gamma coherence, also increases for all values of $s_{en}$ and $\rho _p$ (Fig. 7d, e).

**Fig. 8:**
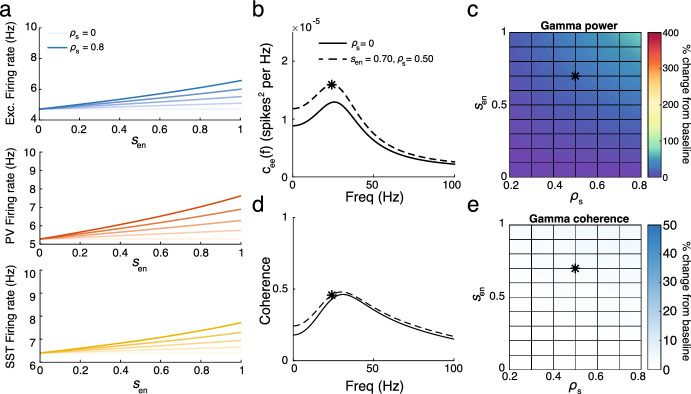
**a** Firing rates of excitatory (top), PV (middle) and SST (bottom) neurons for a range of ensheathment strengths and probability of ensheathment of PV synapses ($\rho _{s}= 0, 0.2, 0.4, 0.6, 0.8$ from light to dark shades). **b** Power spectrum for the excitatory population for $\rho _{s}$ (solid) and 0.5 (dashed) for $s_{en} = 0.7$. The star indicates the gamma power. **c** The percentage change in gamma power from baseline ($\rho _{s} = 0$) for a range of ensheathment strengths and values $\rho _{s}$. **d**: The same as panel **b** but for coherence. **e** The same as panel **c**, but for gamma coherence (Color figure online)

Figure 8 shows the results for the ensheathment of SST neurons. Similar to the previous findings, there is a reliable increase in firing rates across the three populations, indicating that the ensheathment of SST neurons is sufficient to drive the network into a hyperactive state (Fig. 8a). However, the results for the power spectrum and coherence differ significantly (Fig. 8b–e). The increases in the power spectrum are much more moderate, with gamma power not even doubling for the most extreme values of $s_{en}$ and $\rho _s$. Furthermore, the coherence changed only slightly as $s_{en}$ and $\rho _s$ increased. Overall, while the qualitative increases observed in Sect. 3.1 (particularly those found in Fig. 6) could be achieved with the ensheathment of PV neurons alone, the corresponding increases in the power spectrum and coherence measures would not be replicated with the ensheathment of SST neurons alone.

## Conclusion and Discussion

As experimental techniques continue to advance and uncover additional heterogeneities within neuronal (and non-neuronal) sub-populations, simplified mean-field approximations will continue to be essential for exploring increasingly expansive parameter sets and highlighting key components behind complex processes. In this work, we present such an approximation derived from linear response theory that captures the effects of glial ensheathment on synapses. This theory successfully reproduces recent experimental findings, demonstrating that the network enters a hyperactive state when glial ensheathment of inhibitory synapses is present. It also enables us to examine not only average firing rates but also higher-order network statistics, leading to the testable prediction that both gamma power and gamma coherence significantly increase in the presence of glial ensheathment of inhibitory synapses. Further parameter exploration emphasized the importance of considering more than just average firing rates and reinforced the hypothesis of a division of labor across inhibitory subtypes (Bos et al. 2020). While the ensheathment of PV *or* SST interneurons was sufficient to increase the network’s firing rate, only the ensheathment of PV interneurons led to significant increases in gamma power and gamma coherence.

Glial ensheathment is a dynamic process that remains under investigation. In this work, we focused on the findings of Haruwaka et al. (2024), which observed significant increases in microglial ensheathment of inhibitory synapses before and after anesthesia induced by the administration of isoflurane. This represents a substantial disruption to the system, as firing rates drop considerably in the anesthetized state. These results support the idea that microglia dynamically respond to the low-activity network state by attempting to boost excitatory firing rates, resulting in a rebound effect that manifests as hyperactivity during the emergence from anesthesia. Our theoretical prediction of increased gamma power and gamma coherence (along with a general increase across the power spectrum) during this emergence suggests additional, unintended consequences brought on by increased ensheathment. Since neuronal correlations play a key role in how the brain encodes information and performs computations (Cohen and Maunsell 2009; Cohen and Kohn 2011; Ruff and Cohen 2014; Hazon et al. 2022; Kohn et al. 2016), it will be interesting to see if future experimental studies observe similar increases in glial ensheathment in other brain states with sustained aberrant firing rates, and whether such spurious correlations lead to cognitive impairments. If so, our prediction that PV interneurons are largely responsible for the observed increase in the power spectrum suggests a potential therapeutic avenue—specifically targeting those synapses to release them from glial ensheathment.

These results arise from only one possible effect of glial ensheathment, namely the shielding of the post-synaptic terminal via the clearance of neurotransmitters from the synaptic cleft. This effect is likely shared by two types of glial cells, microglia and astrocytes, and our model does not differentiate between the two. However, there are other pathways through which these cells can impact network dynamics, including synaptic pruning, adjustment of resting potentials via ion buffering, and the release of gliotransmitters (Paolicelli et al. 2011; Larsen et al. 2014; Covelo and Araque 2018). The strength of these additional pathways will likely depend on the proximity of the glial cell to the synapse. Future work should aim to extend the microscale model presented here to account for these mechanisms and provide a more complete view of an “effective” glial cell. We anticipate that the mean-field approximation derived here can be extended to account for these additional pathways. For example, in the case of the reversal potential, once the effect of ensheathment is understood, the corresponding parameter $E_L$ could be extended as a function of $s_{en}$ and incorporated into the theory in a similar fashion to how we treated synaptic strength and time constants.

Our mean-field approximation (Eq. 14) relied on numerical computations to estimate the steady-state firing rate, the linear response function for the neuron subclasses, and the power spectrum of the unperturbed system. Analytical expressions for these functions have been derived for simplified leaky integrate-and-fire neurons, but they rely on non-intuitive mathematical functions like the complementary error function and parabolic cylinder functions (Holden 1976; Lindner and Schimansky-Geier 2001; Lindner et al. 2002). While we lack such analytical expressions, the exponential integrate-and-fire neuron used here offers the benefit of a more biologically realistic spiking mechanism, while remaining computationally efficient for our spiking neuronal network. We also left the mean-field approximation in matrix form, as its expansion would be largely uninterpretable. While concise formulas can be derived for simplified networks consisting of one or two populations of neurons (Lindner et al. 2005), this task becomes significantly more complex as the number of populations increases. This complexity also constrained our parameter search in Sect. 3.3 to only consider binary levels of ensheathment-not due to computational concerns, but rather due to the difficulty of visualizing and interpreting results in an ever-growing parameter space, only some of which may be biologically realistic. Nevertheless, our theoretical framework allowed us to explore a relatively large parameter space of the underlying system, revealing a non-intuitive result. Moving forward, we aim to expand our parameter space exploration in collaboration with experimental partners to pursue the most biologically relevant directions.

Furthermore, our theory yields results that deviate from a naïve mean-field approach, which could guide future mean-field models considering similar types of heterogeneity. However, while the results hold for an arbitrary number of discrete levels of glial ensheathment strengths, further work is needed to address the more realistic scenario in which these strengths follow a continuous distribution. Indeed, as suggested by the microscale model and the corresponding linear fit shown in Fig. 1, there are no clear thresholds separating ‘low contact’ from ‘high contact’. As a result, binning glial ensheathment into discrete levels is an oversimplification, both biologically and, consequently, within the model. If experimental data can be collected to describe this continuous distribution, there is potential for a straightforward adaptation of the mean-field methods presented here to account for it. Specifically, the discrete sums averaging the level of glial ensheathment in our equations (e.g., Eq. 8, Eq. 12) could be replaced with integrals over the corresponding probability density function. However, additional work is required not only to make this argument more rigorous but also to conduct further simulations to demonstrate that such an approximation remains appropriate.

Overall, this work provides a foundation for understanding how the heterogeneities introduced by glial ensheathment influence network dynamics and offers a framework for future investigations that examine the role of glial cells in shaping complex neural computations.

## Data Availability

The code to reproduce the main figures of the paper, along with demos for running the simulations and theoretical analysis, is available in a publicly accessible Zenodo repository (10.5281/zenodo.15086374).
